# Investigation of respiratory rate in patients with cystic fibrosis using a minimal-impact biomotion system

**DOI:** 10.1186/s12890-022-01855-w

**Published:** 2022-02-11

**Authors:** Svenja Straßburg, Carolin-Maria Linker, Sebastian Brato, Christoph Schöbel, Christian Taube, Jürgen Götze, Florian Stehling, Sivagurunathan Sutharsan, Matthias Welsner, Gerhard Weinreich

**Affiliations:** 1 Department of Pneumology, University Medicine Essen – Ruhrlandklinik, University Duisburg-Essen, Tüschener Weg 40, 45239 Essen, Germany; 2 Information Processing Lab, Faculty of Electrical Engineering, Information Engineering - TU Dortmund, Dortmund, Germany; 3 SWG Sportwerk GmbH & Co. KG, Dortmund, Germany; 4 Center of Sleep and Telemedicine, University Medicine Essen – Ruhrlandklinik, University Duisburg-Essen, Essen, Germany; 5 Pediatric Pulmonology and Sleep Medicine, Cystic Fibrosis Center, Children’S Hospital, University Duisburg-Essen, Essen, Germany

**Keywords:** Cystic fibrosis, Respiratory rate, Telemedicine, Home monitoring, Exacerbation

## Abstract

**Background:**

In this study we tested the hypothesis that in patients with cystic fibrosis (pwCF) respiratory rate (RR) is associated with antibiotic treatment, exacerbation status, forced expiratory volume in one second (FEV1) and C-reactive protein (CRP).

**Methods:**

Between June 2018 and May 2019, we consecutively enrolled pwCF who were referred to our hospital. We determined RR and heart rate (HR) by using the minimal-impact system VitaLog during the hospital stay. Furthermore, we performed spirometry and evaluated CRP.

**Results:**

We included 47 patients: 20 with pulmonary exacerbation and 27 without. RR decreased in patients with exacerbation (27.5/min (6.0/min) vs. 24.4/min (6.0/min), p = 0.004) and in patients with non-exacerbation (22.5/min (5.0/min) vs. 20.9/min (3.5/min), p = 0.024). Patients with exacerbation showed higher RR than patients with non-exacerbation both at the beginning (p = 0.004) and at the end of their hospital stay (p = 0.023). During the hospital stay, HR did not change in the total cohort (66.8/min (11.0/min) vs. 66.6/min (12.0/min), p = 0.440). Furthermore, we did not find significant differences between patients with exacerbation and patients with non-exacerbation (67.0/min (12.5/min) vs. 66.5/min (10.8/min), p = 0.658). We observed a correlation of ρ = -0.36 between RR and FEV1. Moreover, we found a correlation of ρ = 0.52 between RR and CRP.

**Conclusion:**

In pwCF requiring intravenous therapy, respiratory rate is higher at their hospital admittance and decreased by the time of discharge; it is also associated with C-reactive protein. Monitoring RR could provide important information about the overall clinical conditions of pwCF.

## Background

Cystic fibrosis (CF) is an autosomal recessive disease leading to shorter life expectancy. More than 80.000 individuals are affected by CF worldwide [1–3]. The highest prevalence occurs in Europe, North America and Australia [4].

Thanks to continuous medical progress, median survival age increased significantly to above 40 years since the first description of CF in 1938 [4, 5]. Mutations in the cystic fibrosis transmembrane conductance regulator (CFTR) gene influence the conductance of chloride and bicarbonate ions through the cell membrane leading to dehydration of the epithelial liquid film in affected organs. Thus, CF is a multisystem disease. Morbidity and mortality are mainly determined by chronic inflammation and frequent infections of the lungs [6]. CF related bacterial overgrowth is associated with frequent pulmonary exacerbations that require antibiotic treatment. Typical exacerbation symptoms are more frequent cough, increased purulent secretion, fever, weight loss, reduced resilience, decline of lung function and tachypnoea [7].

Previous studies have shown that respiratory rate (RR) can be used as a marker for clinical outcome of various diseases. For instance, in chronic obstructive pulmonary disease (COPD) a correlation between RR and exacerbation-related hospitalizations has been reported [8–10]. Moreover, RR is a predictor for mortality in patients with community-acquired pneumonia [11]. Consequently, RR determination is integrated in common prognosis tools such as the CRB 65 index (confusion, respiratory rate, blood pressure, age >  = 65 years) [12].

In general, there is strong evidence that RR is an important vital sign to predict morbidity and mortality in some lung disorders. Hence, it can be suggested that RR is an important clinical marker for the disease course in patients with cystic fibrosis (pwCF) as well. However, knowledge on the role of RR in CF is scarce. Therefore, in pwCF we tested the hypothesis that RR is associated with antibiotic treatment, exacerbation status, forced expiratory volume in one second (FEV1), and C-reactive protein (CRP).

## Methods

### Patients

Between June 2018 and May 2019 we consecutivley enrolled pwCF who were referred to the Ruhrlandklinik Essen, Germany in order to receive intravenous antibiotic treatment. Hospitalization was indicated due to pulmonary exacerbation or prophylactic antibiotic treatment. The diagnosis of CF was based on the patient’s history and the detection of two disease causing mutations.

Pulmonary exacerbations were determined according to the criteria defined by Fuchs et al. [7]. An exacerbation is present if four of the following criteria are fulfilled and an antibiotic treatment is required: increased secretion, newly occurred haemoptysis, more frequent cough, increasing dyspnoea, fatigue/indisposition, temperature above 38 °C, weight loss, sinus pain or change in sinus discharge, decline of lung function of about 10% and radiological alterations that indicate an infect [7].

For all patients inflammation marker CRP and leucocytes, body temperature and body-mass-index (BMI) were determined on the day of hospitalization. Forced vital capacity (FVC) and FEV1 were determined with a JAEGER MasterScreen Body (CareFusion, Hoechberg, Germany) according to ATS guidelines [11]. We used the Quanjer GLI-2012 regression equations for spirometric indices [13]. Spirometry was performed at the first day.

Referring to the findings of Hart et al. [14] for the correlation between RR and FEV1 we presume ρ = -0.41. Thus, we need a sample size of n = 44 using an alpha of 0.05 and a power of 0.8.

The ethics committee of the University Duisburg-Essen (17-7891-BO) approved the study and all patients provided a written informed consent.

### Biomotion sensor

**Fig. 1:**
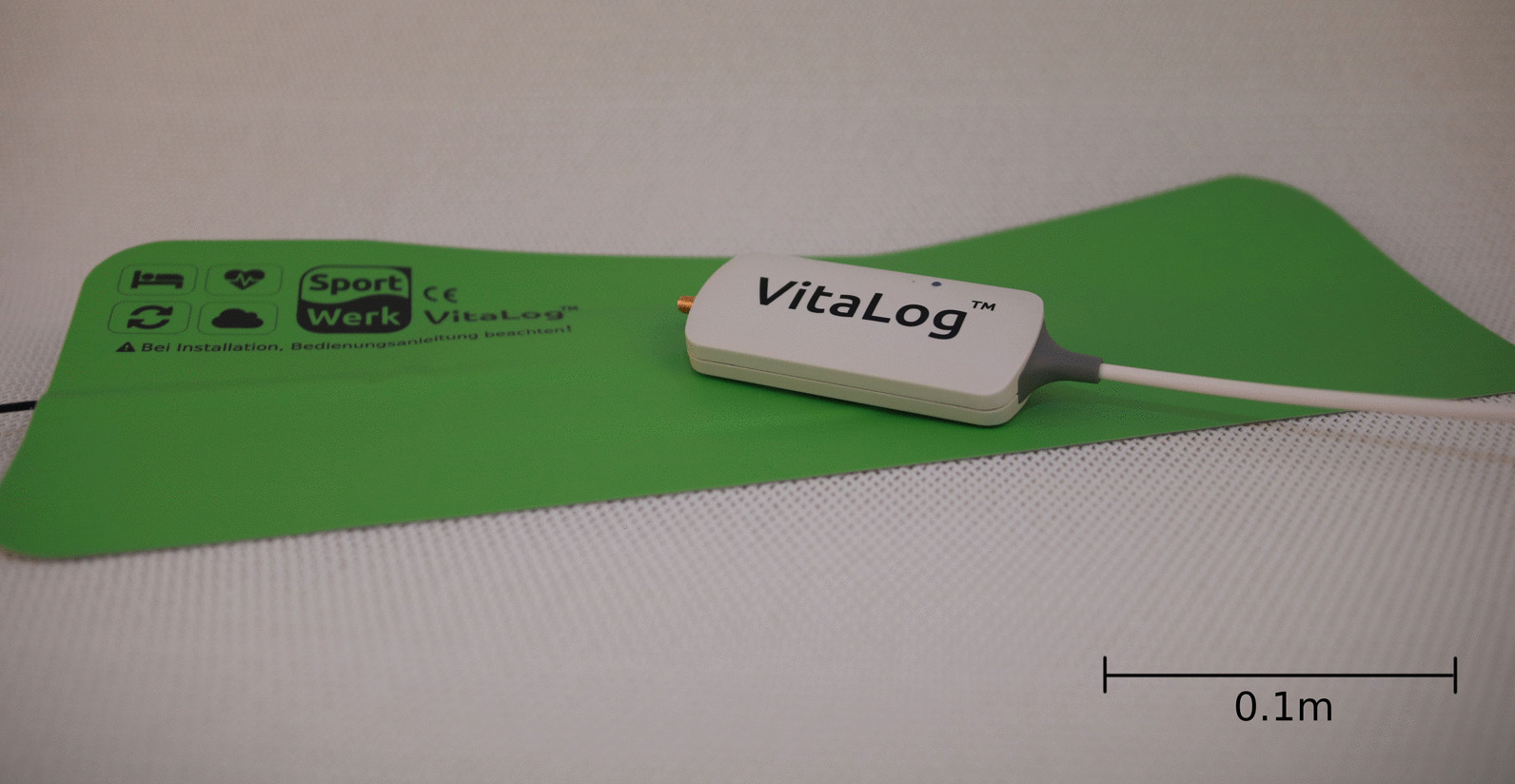
The VitaLog system

We continuously determined RR and heart rate (HR) during night sleep by using the VitaLog (SWG Sportwerk, Dortmund, Germany) system. This device is composed of a sensor and an analysis unit (Fig. 1). The sensor is layed under the bed sheet close to the thorax in order to deviate body movements. Technology is based on the piezoelectric effect leading to a composed movement signal. Due to this minimal-impact setting VitaLog does not interfere with patients’ comfort. The analysis unit is positioned next to the bed. In recent studies our research group evaluated the VitaLog sensor for various vital signs including HR with a very good correlation compared to ECG [15] and RR with a very good correlation compared to nasal flow [16].

### Statistics

Data are presented as mean (standard deviation). Mann–Whitney-U-test was applied to test differences between subgroups and Wilcoxon signed rank test was performed to test differences during treatment. Uncertainty was reported using 95% confidence interval (CI). Fisher’s exact test was applied for categorical variables. Correlation analysis was performed using Spearman correlation coefficients (ρ). We used box plots in order to present the variability of RR and HR. All statistics were performed with Matlab2020b (MathWorks®, Natick, Massachusetts, USA). A P-value < 0.05 was considered statistically significant.

## Results

We included 47 patients. In general, our study group was young, mainly male and had several comorbidities (Table 1). Our study cohort consisted of patients who received prophylactic antibiotic treatment in hospital 57% (27/47) and patients suffering from an acute exacerbation 43% (20/47). Age ranged from 18 to 59 y, BMI from 11.0 to 32.3 kg/m^2^, FEV1 from 17.9 to 81.0%, FVC from 18.0 to 88.7%, Leukocyte from 3.7*10^9/l to 15.3*10^9/l, and CRP from 0.1 to 27.4 mg/dl. Length of hospital stay ranged from 1 to 18 days.

**Table 1:** Patient characteristics at baseline

	All (n = 47)	Exacerbated (n = 20)	Non-Exacerbated (n = 27)
Age, y (SD)	30.7 (10.4)	27.9 (8.9)	32.7 (11.0)
BMI, kg/m^2^ (SD)	20.4 (3.8)	18.5 (3.5)*	21.8 (3.5)
*Sex*			
Female, n (%)	20 (43)	9 (45)	11 (41)
Male, n (%)	27 (57)	11 (55)	16 (59)
*Genotype*			
F508del homozygous, n (%)	23 (49)	11 (55)	12 (45)
F508del heterozygous, n (%)	16 (34)	7 (35)	9 (33)
Other, n (%)	8 (17)	2 (10)	6 (22)
FEV_1_, L (SD)	1.48 (0.69)	1.11 (0.39)*	1.74 (0.74)
FEV_1_, % predicted (SD)	38.0 (15.5)	28.4 (7.9)*	45.2 (15.9)
FVC, L (SD)	2.32 (1.06)	1.76 (0.59)*	2.72 (1.15)
FVC, % predicted (SD)	50.0 (17.1)	38.7 (9.0)*	57.9 (17.1)
Leucocyte, 10^9/l (SD)	8.9 (3.1)	10.9 (3.0)*	7.5 (2.2)
CRP, mg/dl (SD)	3.5 (5.3)	6.9 (6.7)*	1.0 (0.8)
Pancreatic insufficiency, n (%)	43 (91)	18 (90)	25 (93)
*P. aeruginosa* positive, n (%)	33 (70)	13 (65)	20 (74)
Cystic fibrosis-related diabetes, n (%)	16 (34)	7 (35)	9 (33)
O2-Supplementation, n (%)	21 (45)	14 (70)*	7 (26)
NIV, n (%)	7 (15)	6 (30)*	1 (4)
t_VitaLog_ (days) (SD)	6.4 (3.7)	7.6 (4.1)	5.5 (3.2)

NIV: non invasive ventilation

*p < 0.05 between subgroups

**Fig. 2:**
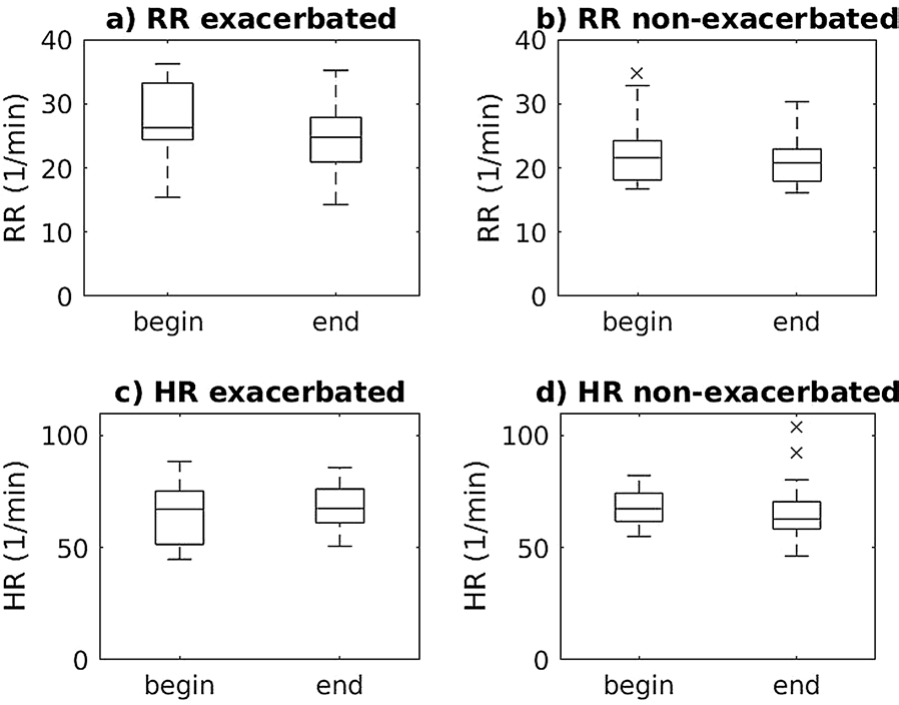
RR (**a** and **b**) and HR (**c** and **d**) changes during hospital stay for exacerbated (**a** and **c**) and non-exacerbated (**b** and **d**) pwCF. The lowest point on the box-plot describes the minimum value and the highest point the maximum value of the analyzed data. The box is drawn from the lower quartile (Q25) to the upper quartile (Q75) with a horizontal line in the middle to label the median

RR ranged from 14.3/min to 36.2/min (n = 43). At the end of the hospital stay we observed a decrease of RR in the total cohort (24.6/min (5.9/min) vs. 22.4/min (5.0/min), p < 0.001) meaning a drop of − 2.2/min (95% CI:  − 3.3\min to − 1.0/min). Moreover, RR decreased in patients with exacerbation (27.5/min (6.0/min) vs. 24.4/min (6.0/min), p = 0.004) meaning a drop of − 3.1/min (95% CI:  − 5.2/min to − 0.9/min) and in patients with non-exacerbation (22.5/min (5.0/min) vs. 20.9/min (3.5/min), p = 0.024) meaning a drop of − 1.5 (95% CI:  − 2.8/min to − 0.2/min) (Fig. 2). Patients with exacerbation (n = 18) showed higher RR than patients with non-exacerbation (n = 25) both at the beginning (p = 0.004) and at the end of their hospital stay (p = 0.023).

HR ranged from 44.8/min to 103.9/min (n = 43). We did not observe any HR changes during the hospitalization (66.8/min (11.0/min) vs. 66.6/min (12.0/min), p = 0.440). For patients with exacerbation (n = 18) we did not observe statistically significant HR changes (65.2/min (14.2/min) vs. 68.8/min (10.5/min), p = 0.184). Of note, patients with non-exacerbation (n = 25) showed a statistically significant decrease in HR (68.0/min (8.2/min) vs. 65.0/min (12.9/min), p = 0.031). However, this decrease is not of clinical relevance. Furthermore, we did not find any significant differences between exacerbated and patients with non-exacerbation (67.0/min (12.5/min) vs. 66.5/min (10.8/min), p = 0.658).

**Fig. 3:**
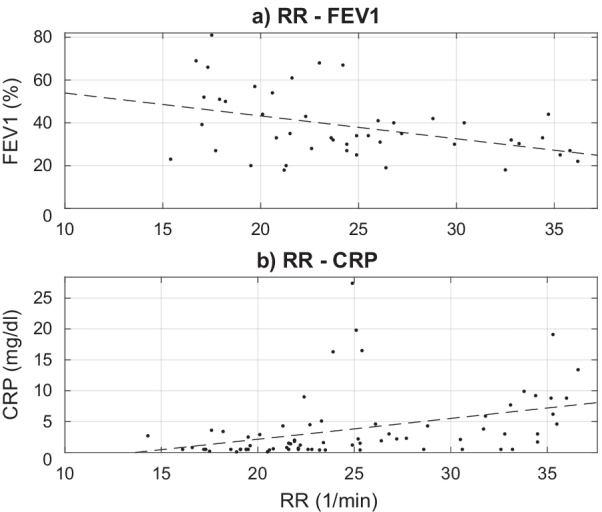
Relationship of RR with FEV1 (**a**) and CRP (**b**), respectively

We found a weak negative correlation ρ = − 0.36 (p = 0.015) between RR and FEV1 measurements (n = 45) as well as a moderate positive correlation ρ = 0.52 (p < 0.001) between RR and CRP measurements (n = 72) (Fig. 3).

## Discussion

With the presented study we provided some novel insights and confirmed observations of previous studies. First, during hospital stay RR is increased in pwCF compared to known RR of the general population. Second, we observed that RR decreased in the course of antibiotic treatment. Third, RR was higher in patients with exacerbation compared to patients with non-exacerbation. Fourth, we found that RR is associated with FEV1. Fifth, HR did not change relevantly during antibiotic treatment.

For healthy adults RR ranges between 12/min and 20/min [17]. In our cohort we observed that only a minority has an RR in the normal range whereas most of our CF patients have increased RR compared to healthy subjects. Previously, it was reported that mean RR was 34/min for adult pwCF with mean FEV1 of 22.5% prior to lung transplantation [18]. However, knowledge on RR in adult pwCF is limited. Thus, evidence does not exist on common RR for this cohort. Therefore, further investigations are required in order to elucidate this research topic including possible clinical implications. Though, a few studies showed that infants and young adults have increased RR compared to healthy peers [14, 19, 20]. Korten et al. observed for pwCF during the first year of life a consistently higher RR of 4/min independent of respiratory infections [19]. Paranjape et al. found significantly increased RR (19.5/min vs. 16.5/min) for pwCF with median age of 9.6 years and median FEV1 of 87% [20]. For clinically stable CF patients with a mean age of 14.2 years and a mean FEV1 of 28.7% Hart et al. observed a mean RR of 22.6/min which is similar to our observations [14]. Hence, our findings of increased RR in adult pwCF are in line with reported elevated RR values in adolescent pwCF.

We could confirm the results reported earlier that RR decreases in the course of antibiotic treatment. Bell et al. found in a smaller cohort of 22 adult pwCF that RR went down from 24/min (on day 1) to 19/min (on day 15) [21]. However, this cohort from 1999 differs to our patient group since treatment progress over the last 20 years had a positive impact on clinical outcome. For instance, the cohort from Bell et al. had a mean age of 23.7 years and a mean FEV1 of 28.5% as opposed to our patient group with a mean age of 30.7 years and a mean FEV1 of 38.0%. Moreover, in patients with non-exacerbation during prophylactic antibiotic treatment we also observed decreasing RR caused by lowering of bacterial load, reduced secretion and therby improved respiration. Thus, RR may be an additional marker for response to antibiotic treatment. This finding provides some new procedures for the monitoring of treatment success beyond the laboratory diagnosis of common clinical infection parameters. Monitoring of RR provides the opportunity to develop a telemedicine-based approach of surveillance in the home environment in order to decrease hospitalization rate.

Evidence suggests that RR is an established risk parameter for hospital mortality for patients with community acquired pneumonia. McFadden et al. noticed that increased RR (mean 29.7/min) indicated pulmonary infections prior to the clinical diagnosis [22]. Furthermore, RR is known to increase during exacerbations of different underlieing pulmonary diseases. For instance, Yanez et al. observed that in COPD patients RR rises significantly days before hospitalization [8]. The authors concluded that continuous RR monitoring may offer a window of opportunity for early intervention.

To our knowledge, this is the first study observing that RR and FEV1 are negatively associated in adult patients. This complements the findings of a previous study. Hart et al. reported that in clinically stable, adolescent pwCF a reduction in FEV1 was accompanied by an increase in RR [14].

In general, data on HR in pwCF is scarce. In contrast to our findings Bell et al. observed that HR decreases were clinically relevant between day 1 and day 8 of exacerbation treatment [21]. Furthermore, mean HR at hospitalization was much higher compared to our cohort (100/min vs. 67/min). As opposed to our study Bell et al. used a short-time single measurement of HR. Thus, it cannot be excluded that at the beginning of the hospital stay the so called white-collar effect may have occurred explaining the different findings. However, as evidence on HR progression during hospital stay is mixed, further studies are necessary in order to gain more profound knowledge on this topic.

Furthermore, we found that RR is associated with CRP. As CRP is a common biomarker for the diagnosis of pulmonary exacerbation [23], but does not always reflect the requirement of antibiotic therapy, it could be suggested that a combination of RR and CRP might be a helpful tool for treatment decision-making. Further studies are needed to elucidate the clinical impact of this approach.

This study has several strengths. First, compared to previous studies we provide a considerably higher sample size. Second, our set-up of long-term monitoring during the hospital stay allows valuable insights in vital parameter trends with clinical implications. Third, we were the first group investigating in long-term continuous RR and HR monitoring of adult pwCF. Fourth, as VitaLog is a simple, reliable and minimal-contact device, RR and HR can be determined with low failure rates.

However, we also acknowledge some limitations. First, in terms of providing common RR and HR values in pwCF we have to admit the bias that only hospitalized patients during treatment were included into our analysis. Second, as we did not adjust for CF-specific features the underlying nature of RR changes is undetermined. Third, we were also only able to determine vital signs during the hospital stay and not until the end of outpatient antibiotic treatment. Therefore, it can be assumed that RR is lower in common non-hospitalized pwCF. Thus, further investigations are required in order to test this hypothesis.

## Conclusion

In pwCF requiring intravenous therapy, respiratory rate is higher at their hospital admittance and decreased by the time of discharge; it is also associated with C-reactive protein. Monitoring RR could provide important information about the overall clinical conditions of pwCF.

## Data Availability

The datasets used in this study are available from the corresponding author upon reasonable request.

## Abbreviations

CF: Cystic fibrosis
CFTR: Cystic fibrosis transmembrane conductance regulator
CI: Confidence interval
COPD: Chronic obstructive pulmonary disease
CRP: C-reactive protein
FEV1: Forced expiratory volume in one second
FVC: Forced vital capacity
HR: Heart rate
pwCF: Patients with cystic fibrosis
RR: Respiratory rate
